# PP08 Prognostic Factors For Survival In Adults With Burkitt Lymphoma: A Systematic Review And Meta-Analysis

**DOI:** 10.1017/S0266462324001818

**Published:** 2025-01-07

**Authors:** Aythami De Armas-Castellano, Diego Infante-Ventura, Yadira González-Hernández, Tasmania del Pino-Sedeño, Beatríz Leon-Salas, Raúl Quirós-López, Mar Trujillo-Martín, EuroBloodNet Working Team

## Abstract

**Introduction:**

Burkitt lymphoma (BL) is a rare and highly aggressive subtype of non-Hodgkin’s lymphoma. Several studies have identified prognostic factors (PFs) for disease progression and mortality among adults with BL. However, there is no consensus on risk stratification based on PFs. This study aims to identify, critically assess, and synthesize the available evidence on PFs for survival in adults with BL.

**Methods:**

A systematic review of the literature was conducted. MEDLINE, Embase, and CENTRAL were searched from inception to 22 February 2022. Randomized or non-randomized clinical trials and longitudinal observational studies were eligible for inclusion. Reference screening, data extraction, and risk-of-bias assessment using the Quality in Prognosis Studies (QUIPS) tool for prognostic factor studies were conducted independently and in duplicate. Publication bias was examined by visual inspection of funnel plots. Effect measures and the corresponding 95% confidence intervals were pooled with an indirect variance estimation in meta-analyses using Review Manager 5, and sensitivity analyses were conducted. Certainty of evidence was assessed using GRADE.

**Results:**

The search identified 1,119 references after duplicate removal. Of these, 76 potentially relevant papers were selected for full-text assessment and 36 studies (N=10,882) reported in 39 articles were eligible for inclusion. Older age, higher performance status, and central nervous system involvement were associated with poorer overall survival (OS) and progression-free survival (PFS). Black patients exhibited significantly lower OS and relative survival. Bone marrow involvement and higher albumin levels were associated with poorer OS. Treatment with rituximab and treatment with methotrexate were associated with better OS and PFS. No significant differences in survival were found for HIV status, sex, and risk stratification.

**Conclusions:**

This study, framed within a collaboration with European Reference Network EuroBloodNet, provides a comprehensive and methodologically rigorous evidence review on PFs in adults with BL. Several significant associations of PFs and survival estimates were observed, providing data to inform treatment decisions and to improve patient care.

